# Inconsistent reproductive isolation revealed by interactions between *Catostomus* fish species

**DOI:** 10.1002/evl3.29

**Published:** 2017-10-27

**Authors:** Elizabeth G. Mandeville, Thomas L. Parchman, Kevin G. Thompson, Robert I. Compton, Kevin R. Gelwicks, Se Jin Song, C. Alex Buerkle

**Affiliations:** ^1^ Department of Botany and Program in Ecology University of Wyoming Laramie Wyoming 82071; ^2^ Department of Biology University of Nevada Reno Nevada 89557; ^3^ Colorado Parks and Wildlife Montrose Colorado 81401; ^4^ Wyoming Game and Fish Department Laramie Wyoming 82070; ^5^ Department of Ecology and Evolutionary Biology University of Colorado Boulder Colorado 80309

**Keywords:** Admixture, ancestry, *Catostomus*, hybridization, introduced species, reproductive isolation

## Abstract

Interactions between species are central to evolution and ecology, but we do not know enough about how outcomes of interactions between species vary across geographic locations, in heterogeneous environments, or over time. Ecological dimensions of interactions between species are known to vary, but evolutionary interactions such as the establishment and maintenance of reproductive isolation are often assumed to be consistent across instances of an interaction between species. Hybridization among *Catostomus* fish species occurs over a large and heterogeneous geographic area and across taxa with distinct evolutionary histories, which allows us to assess consistency in species interactions. We analyzed hybridization among six *Catostomus* species across the Upper Colorado River basin (US mountain west) and found extreme variation in hybridization across locations. Different hybrid crosses were present in different locations, despite similar species assemblages. Within hybrid crosses, hybridization varied from only first generation hybrids to extensive hybridization with backcrossing. Variation in hybridization outcomes might result from uneven fitness of hybrids across locations, polymorphism in genetic incompatibilities, chance, unidentified historical contingencies, or some combination thereof. Our results suggest caution in assuming that one or a few instances of hybridization represent all interactions between the focal species, as species interactions vary substantially across locations.

**Impact Summary**

Species occupy variable environments over large geographic areas, where they interact with a range of other species, including closely related species. Outcomes of interactions between species can vary across locations, depending on factors like the environmental context and which other species are in close proximity. While ecological components of species interactions are known to vary substantially, many studies of evolution employ a simplifying assumption that evolutionary processes are relatively consistent in all places where a pair of species interacts. In this article, we considered how reproductive isolation between species (the mechanisms that keep species from interbreeding with closely related species) varies when the same pairs of species interact repeatedly in a range of environments. We used large genetic datasets to study six species of *Catostomus* fish (“suckers”) in the US mountain west. From previous work, we know that these species sometimes interbreed, producing hybrid individuals. In this study, we found that occurrence and extent of hybridization vary dramatically across many replicate locations where species come into contact. This pattern holds across multiple pairs of species. From these results, we conclude that these fish species are maintained as separate species (prevented from interbreeding) by different factors in different locations, with variable effectiveness of barriers to reproduction between different species. If these fish species are typical, this means that evolutionary biologists might need to incorporate a greater expectation of geographic variation into studies of evolutionary processes like hybridization. Our results are also extremely applicable to conservation of native *Catostomus* fish species that are threatened by hybridization, since this research suggests that successful conservation strategies might also need to be tailored to individual rivers.

Species interactions can produce variable ecological outcomes (e.g., Brooks and Dodson 1965; Paine 1966; Carpenter and Kitchell 1988; Valone and Brown 1995; Brown et al. 2001), such that contingency and variability are commonly expected, even if deterministic processes also contribute (Hubbell 2001; Jackson et al. 2009). While genetic and phenotypic variance is a central subject of study in evolutionary biology, simpler, more deterministic models are appealing and are often assumed to apply to the history of organisms and the evolution and genetics of their traits (Weiss 2008; Hewitt 2011; Rockman 2012). Theory predicts and empirical studies show that species' histories and trait architectures are partly, and sometimes largely, idiosyncratic, so some combination of contingency and determinism is a plausible expectation in evolutionary genetics (Taylor and McPhail 2000; Losos 2010; Kaeuffer et al. 2012; Rockman 2012; Soria‐Carrasco et al. 2014). Our study characterized the extent to which species interactions, in this case hybridization, result in consistent outcomes as predicted by simple models of reproductive isolation between species, and to what extent hybridization is variable as suggested by empirical examples.

Simple models for isolating barriers have played a central role in the conceptualization of speciation (e.g., two locus Dobzhansky‐Muller incompatibilites) and in some, possibly exceptional, cases have empirical support from trait mapping (Rieseberg and Blackman 2010; Wolf et al. 2010; Nosil and Schluter 2011; Yuan et al. 2013). Despite the appeal of simple models, isolation between species is expected to arise from multiple, polygenic traits that are expressed at multiple stages of the life history (Ramsey et al. 2003; Lindtke et al. 2014). As is true for most quantitative traits, one would expect the relevant phenotypes to be shaped by functional genetic polymorphisms that vary across a species' range and to be influenced by environmental variation. Additionally, similar phenotypic outcomes of independent evolution might be evident (Losos 1998; Mahler et al. 2013), but develop as a result of different underlying mutations (Natarajan et al. 2016) or processes (Stayton 2015). Consequently, the genetics of speciation and dynamics of incompletely isolated species should vary among locations where species potentially hybridize. But this variance has rarely been quantified across several natural populations. If appreciable variation exists in the evolutionary outcomes of contact between species, these species are unlikely to be isolated as a result of traits with simple genetic architectures that are shared and independent of environment across the species' ranges. Instead, in these cases, speciation and reproductive isolation are more contingent, as a result of variable genetics or environments in which traits are expressed.

Our previous work on hybridization among *Catostomus* fishes demonstrated how reproductive isolation can vary geographically (Mandeville et al. 2015). We found substantial differences in the outcomes of hybridization in 785 fish from three sites, involving five different parental species (Mandeville et al. 2015). There were hybrid fish in each of the three rivers, but the species that produced hybrid offspring varied by river, as did the extent of hybridization and backcrossing. If we had sampled any one of these rivers in isolation, we would have been misled about the dynamics of reproductive isolation for these species (as was initially the case for McDonald et al. 2008). With samples from only three sites, we established that there was variability, but we were not able to characterize the extent and nature of variation in reproductive isolation and outcomes of hybridization.

To accurately characterize species interactions and hybridization, it was necessary to work at a broader spatial scale, with informative genomic data. In this study, we employed extensive geographic and taxonomic sampling to determine the consistency of species interactions. Our primary goal was to measure variation in hybridization among *Catostomus* fishes in Wyoming and Colorado on a broad geographic scale, using genomic data to estimate ancestry and distinguish among distinct hybridization outcomes. Our study addressed two specific questions: **(1)** To what extent are outcomes of interactions between *Catostomus* species consistent across sites and rivers?, and **(2)** What relationships exist between intraspecific genetic variation and outcomes of contact and hybridization between species? Broad geographic and genomic sampling allowed us to characterize hybridization in an unusually comprehensive manner, leading to greater understanding of how hybridization outcomes vary in natural populations across the shared range of the hybridizing species. We sought to detect the consequences of variation in traits affecting reproductive isolation (Sweigart et al. 2007; Good et al. 2008; Cutter 2012; Kozlowska et al. 2012) and to learn how ecological opportunity, historical contingency, and chance contribute to the evolution and maintenance of reproductive isolation (Taylor and McPhail 2000; Seehausen 2007; Wagner et al. 2012).

## Methods

**Figure 1:**
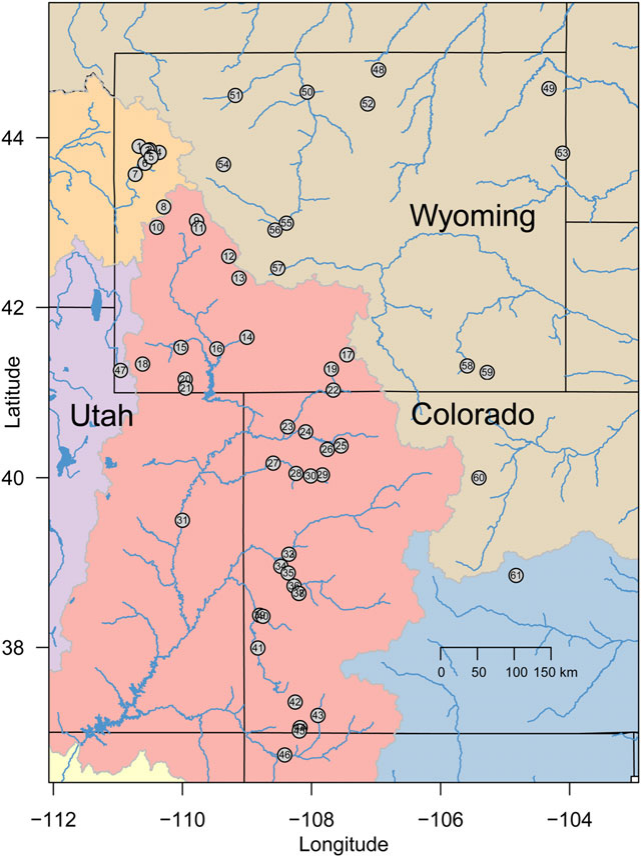
Map showing approximate sampling locations for populations of fish in this study. Boundaries between major river basins are shown in gray, and each major river basin is shown with a different background color. Our study of hybridization focused on the Upper Colorado River basin (UCRB; pink); other populations were sampled for reference individuals of species that were introduced in the UCRB but are native to adjacent basins. For clarity, only major rivers are shown and small streams are omitted.

Sampling for this project was accomplished through partnerships with state and federal agencies. Fin tissue was sampled nonlethally from 2932 individual fish. Samples span six species and hybrids, and represent 61 locations in the US mountain west (Fig. 1; 765 individuals originally collected for McDonald et al. (2008) and Mandeville et al. (2015) were resequenced for this study). Where possible, 20–30 individuals of each species or cross were sampled in each location. Fish were identified phenotypically by experienced field personnel, and fin clips were stored in ethanol. Six species were sampled, including *Catostomus latipinnis* (flannelmouth sucker), *C. discobolus* (bluehead sucker), *C. commersoni* (white sucker), *C. platyrhynchus* (mountain sucker), *C. catostomus* (longnose sucker), and *C. ardens* (Utah sucker), and hybrids of these species. *C. latipinnis*, *C. discobolus*, and *C. platyrhynchus* are native to the Upper Colorado River basin, our primary study area. The other three species are native to adjacent basins, and have been introduced to the Upper Colorado River basin, probably within the past 100 years (Baxter et al. 1995; Gelwicks et al. 2009). One introduced species, *C. commersoni*, has become extremely widespread and abundant in its introduced range (Gill et al. 2007; Gelwicks et al. 2009); the other two were only sampled in a few locations in the Upper Colorado River basin.

We extracted DNA from fin clips using DNeasy 96 Blood & Tissue Kits (Qiagen, Inc.). We then prepared reduced‐complexity genomic libraries for high throughput DNA sequencing, following the genotyping‐by‐sequencing method in Parchman et al. (2012). For this project, each lane of sequencing included 300–400 individual fish. High throughput DNA sequencing (Illumina Hiseq 2500, SR 1× 100) was completed at the University of Texas, Austin, by the Genome Sequencing and Analysis Facility (UT‐GSAF). Prior to sequencing, UT‐GSAF used a BluePippin (Sage Science) device to size‐select DNA fragments 250–300 base pairs in length from our libraries.

### FILTERING AND ASSEMBLY

DNA sequencing on eight Illumina Hiseq 2500 lanes produced 427 gigabytes of raw data, representing 1.77× 10^9^ short DNA sequences. We filtered the data to remove common contaminant sequences (PhiX, *E. coli*) and excess Illumina primers and adaptors, and retained 1.43× 10^9^ reads. We then used a custom perl script to match barcode sequences to individual fish, and retained 1.37× 10^9^ sequence reads.

Since there is no draft genome sequence for *Catostomus* species, we constructed an artificial reference genome using a *de novo* assembly (as in Parchman et al. 2012; Gompert et al. 2014; Mandeville et al. 2015). We used smng (SeqMan NGen, DNAstar, Inc.) to assemble a subset of the data (40 million reads). We required a minimum match percentage of 90% and a depth of at least 25 reads to retain a contig. This *de novo* assembly produced an artificial reference genome with 327,388 contigs. We then completed a reference‐based assembly of all sequence data using bwa version 0.7.5 (Li and Durbin 2009). 9.38× 10^8^ reads (68% of barcoded reads) assembled to the reference. To ensure that all individuals had sufficient data for downstream analyses, we removed 147 individuals with fewer than 9845 reads assembled, corresponding to the 5% quantile of assembled reads. We retained 2785 individuals.

### VARIANT CALLING

From the individual assembly files (bam files), we identified single nucleotide variants using samtools and bcftools (version 0.1.19; Li et al. 2009; Li 2011). We required that >70% of all individuals (>1950 individuals) had data at a genetic site to identify a variable nucleotide and calculate genotype likelihoods at that locus. We initially identified 100,242 single nucleotide variants. To compare multiple species, we wanted sites that were polymorphic in multiple populations, so we excluded sites with minor allele frequencies <5% (Gompert et al. 2014). We also excluded sites with more than two alleles. We ensured greater independence of loci by randomly selecting one variable site per contig. We retained 11,221 SNPs that we used for all analyses of hybridization.

### ANCESTRY ESTIMATION WITH ENTROPY

We used a hierarchical Bayesian model, entropy (Gompert et al. 2014), to estimate ancestry of each individual. Like structure (Pritchard et al. 2000; Falush et al. 2003), entropy does not require *a priori* information about membership of individuals in species or demes. We distinguished F_1_, backcrossed, and advanced generation hybrids by combining estimates of *q* and *Q* from entropy, where *q* is the proportion of an individual's ancestry from each parental species, and *Q* is the proportion of loci in an individual that have ancestry from both parental species.

To estimate *q* (proportion of ancestry) we ran a model with k=6 genetic clusters (one for each species; McDonald et al. 2008; Mandeville et al. 2015), using data at 11,221 loci. entropy uses Markov Chain Monte Carlo (MCMC) to estimate posterior distributions for all parameters. We ran three MCMC chains for 45,000 steps, retaining every 5th step, and discarded the first 40,000 steps as burn‐in. This resulted in 1000 samples from the posterior distribution of each of three chains. For a subset of individuals, we plotted MCMC chains to check for adequate mixing and convergence of parameter estimates. We estimated posterior distributions for proportion of ancestry (*q*) in each fish and genotype at each locus for each individual.

We also estimated interspecific ancestry (*Q*), the proportion of loci in an individual with ancestry from both parental species, using entropy (Gompert et al. 2014). Estimates of *Q* are particularly informative about whether hybrids are the progeny of a cross involving the parental species, or are advanced generation hybrids. Interspecific ancestry is expected to be Q=1 for F_1_ hybrids (each locus has one allele copy from each parental species), and has an expected value of Q=0.5 for F2 hybrids and backcrosses between F_1_ hybrids and parental species. To achieve model convergence and to ensure that relevant local parental allele frequencies were used for local hybrids, we ran entropy separately for each hybridizing species pair in each river where >3 individuals of the same hybrid cross were sampled, using all 11,221 SNPs. For each hybrid cross in each river, we ran entropy for 200,000 steps with a k=2 model and the *Q* model for admixture, discarded the first 150,000 steps as burn‐in, and retained every 10^*th*^ step, resulting in 5000 samples from the posterior distribution for each of three chains. We confirmed mixing and convergence using plots of MCMC chains for a subset of individuals and parameters. We then used the bivariate relationship of *Q* and *q* to characterize the composition and ancestry of hybrids.

### EXTENT OF HYBRIDIZATION

Using estimates of *q* and *Q*, we quantified extent of hybridization for the 22 rivers in the Upper Colorado River basin for which we had sample sizes of >20 individuals and at least one hybrid cross was present. Extensive hybridization occurs in two ways in this system, and we used two different measures to compare across rivers. In some locations, many distinct hybrid crosses (combinations of different parental species) were present; in other locations, extensive hybridization involved backcrossing to parental species. Since hybrid crosses present varied among rivers, our first response variable was the number of distinct hybrid crosses sampled in a location. We measured extent of backcrossing quantitatively by estimating the 95% quantiles of ancestry estimates (*q*) for individual hybrid fish in each tributary where flannelmouth× white hybrids (the most geographically ubiquitous cross) were sampled.

### POPULATION GENETIC AND ENVIRONMENTAL PREDICTORS OF HYBRIDIZATION OUTCOMES

We sorted individuals to species based on entropy results (>95% ancestry in a single genetic cluster, where each genetic cluster corresponds to a species), and identified variable genetic sites independently within each species. We used samtools and bcftools (version 0.1.19) to identify variants, and required that >80% of individuals within a species had data at a site for a single nucleotide variant to be called. We filtered out extremely low frequency variants (present in fewer than three individuals) and selected one SNP per contig. We then ran entropy for each species independently, for k=1–5 genetic clusters. We used posterior estimates from entropy to reestimate genotype at each locus and built a genotype covariance matrix among individuals of each species, which we used in a principal components analysis (prcomp in R, R Development Core Team 2016). We then used PC scores for each species to examine the relationship between intraspecific variation in parental species and outcomes of hybridization.

We also used publicly available data to examine possible correlations (cor.test in R) between extent of hybridization in a location and environmental variables. We used data on land use, elevation, gradient, and other potentially relevant site characteristics, and tested for correlations between these variables and extent of hybridization at a location, as measured by both degree of backcrossing and number of distinct hybrid crosses. A more detailed description of this analysis is in the Supplemental material.

## Results

DNA sequencing produced 1.77× 10^9^ reads. From these data, we identified 11,221 well‐supported independent SNPs for 2785 individual fish. Mean sequence coverage for retained SNPs was 6.1 reads per locus per individual. We then sorted individuals by species, and identified polymorphic loci within each of six parental species (7672–19,797 SNPs per species) to describe the relationship between within‐species genetic structure and hybridization outcomes.

### HYBRIDIZATION PRODUCES DIVERSE OUTCOMES

Our analyses confirm that hybridization among *Catostomus* species is geographically widespread and variable in the Upper Colorado River basin. Of the 61 locations sampled, 38 were within the Upper Colorado River basin, while the other 23 were in adjacent river basins (Fig. 1). Populations outside the basin were sampled to provide reference populations for focal taxa independent of hybridization in the Upper Colorado River basin. For each individual fish, we estimated *q*, the proportion of ancestry in each genetic cluster in a k=6 model using entropy (Gompert et al. 2014). Under this model, each cluster corresponds to a named species (Mandeville et al. 2015).

**Figure 2:**
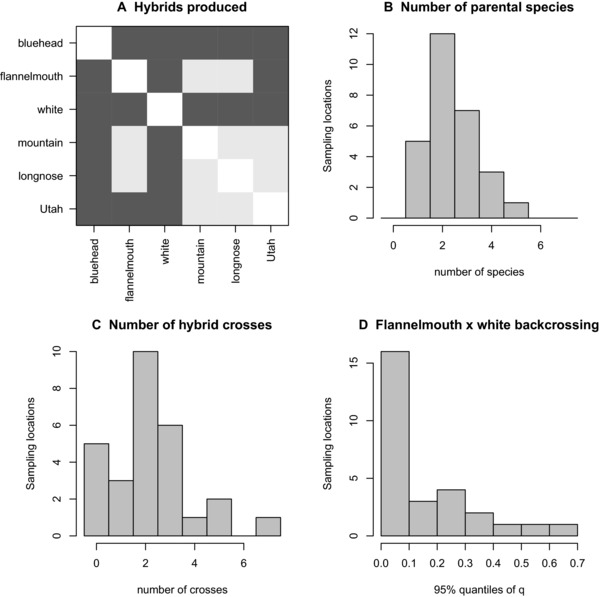
The identity of hybrid crosses (**A**; dark gray–present, light gray–absent), number of parental species (**B**) and number of hybrid crosses (**C**) varies across the 28 rivers in the Upper Colorado River where more than 20 individuals were sampled. Extent of backcrossing in flannelmouth× white hybrids, the most geographically widespread cross, also varies by river (**D**).

**Figure 3:**
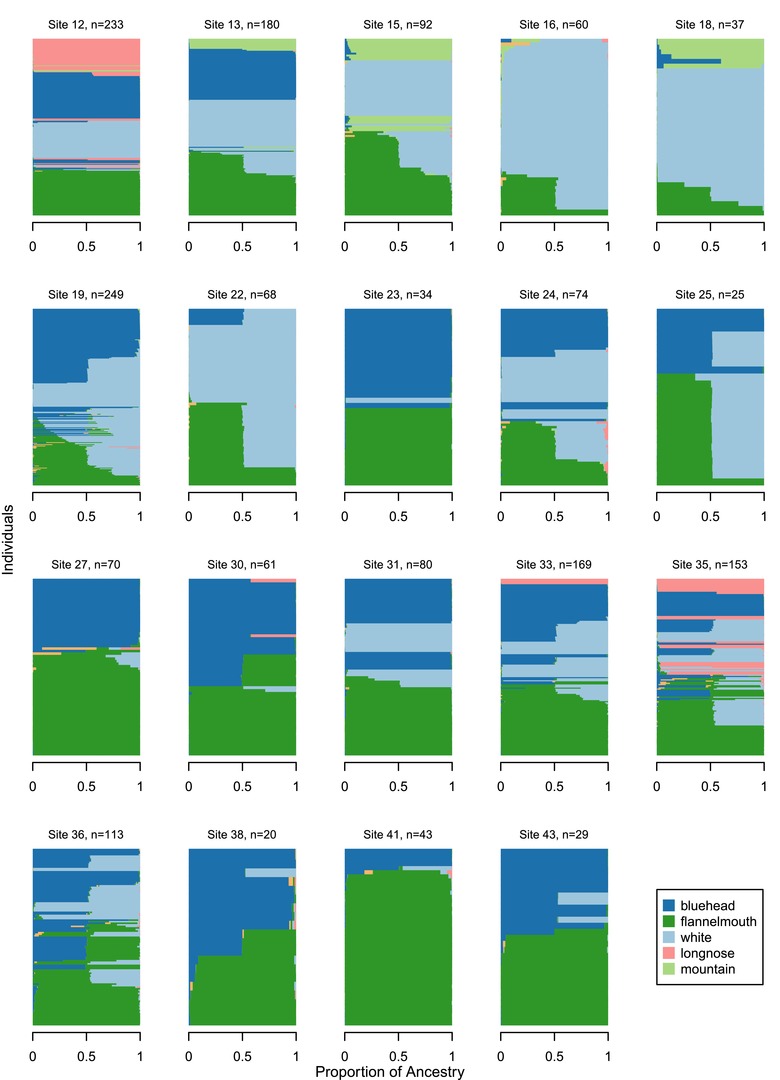
Outcomes of hybridization vary across locations where native and nonnative *Catostomus* species coexist in the Upper Colorado River basin. These 19 plots represent rivers in the Upper Colorado River basin with large sample sizes and multiple species of interest. Individual fish are arranged along the vertical axis of each plot, and bars are colored according to the proportion of an individual's ancestry contributed from each of six possible parental species (entropy, k=6 model; Utah suckers were not sampled in these rivers and are excluded from the legend). Rivers are presented in north to south order.

**Figure 4:**
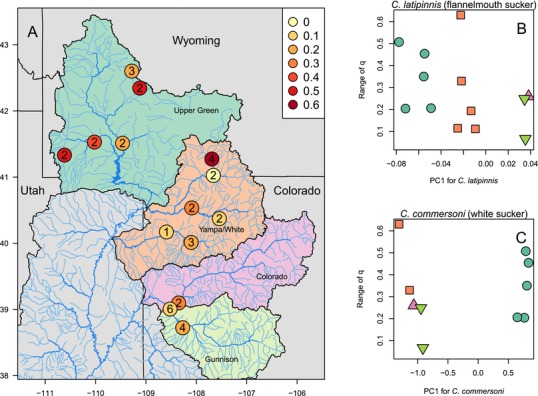
Hybridization outcomes are variable across geographic space (**A**). Within each river basin (color‐coded) where flannelmouth and white suckers come into contact, there are instances of extensive hybridization and more constrained outcomes. Color of points corresponds to the range of q, or proportion of ancestry, in flannelmouth x white hybrids. The text inside the points gives the number of different hybrid combinations in each river. **B, C** There is no correlation between the dominant axis of genetic variation within parental species and hybridization outcomes. For **B** and **C**, the horizontal axis shows population means on PC1, the first principal component of genetic variation, for each parental species. The vertical axis shows the range of *q* values in a river (range between 2.5 and 97.5% quantiles of estimates of *q* for individuals in a population), which is a measure of how much backcrossing occurs in flannelmouth× white hybrids in a river. Points are colored according to river basin in which a sampling location lies.

In 21 out of 28 locations within the Upper Colorado River basin with sample sizes of >20 individuals, we identified at least one type of interspecific hybrid (Fig. 2), but the identity of crosses, the number of crosses, and the extent of backcrossing and later‐generation hybridization varied by river (Fig. 3 and 4). Hybrids were produced between native and nonnative species, but also between pairs of native species. Among the three most common species, bluehead, flannelmouth, and white suckers, we observed more geographic instances of hybridization and more hybrid individuals in the crosses involving nonnative white suckers (*C. commersoni*). Flannelmouth× white sucker hybrids were observed at 15 out of the 28 sites in Figure 2 (246 total individuals), and bluehead× white hybrids were observed at 12 out of those 28 sites (146 total individuals). In contrast, bluehead× flannelmouth hybrids, which involve two native parental species, were observed at 8 of the 28 sites (56 total individuals).

We observed 12 different hybrid crosses among six parental species, including hybrids that occurred outside of the Upper Colorado River basin. To guard against inferring erroneous patterns of hybridization due to model uncertainty, this count only includes crosses for which more than one putative hybrid individual was sampled. Two types of hybrids had ancestry from three parental species (bluehead× flannelmouth× white, Muddy Creek, Colorado River, Gunnison River; flannelmouth× Utah× white, Halfmoon Lake). Of the ten crosses with two parental species, six feature a nonnative species hybridizing with a native species, while four are between two native species. Nine crosses were in the Upper Colorado River basin.

### EXTENT OF HYBRIDIZATION IS VARIABLE ACROSS SPECIES AND LOCATIONS

**Figure 5:**
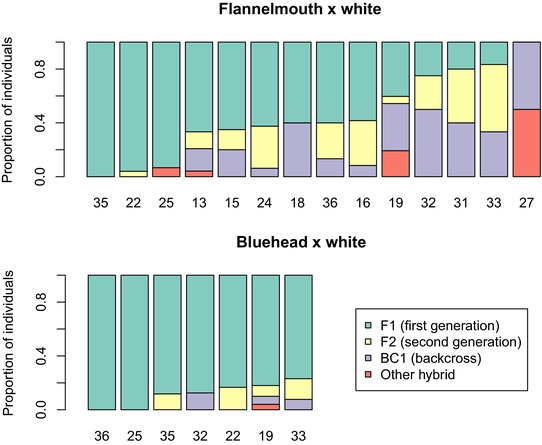
Proportion of hybrids that are likely F_1_, F_2_, and backcrosses varies by cross, and by river within each cross. Stacked bars show the proportion of hybrid individuals in each hybrid category in each river where the two most common crosses were sampled. Both bluehead× white and flannelmouth× white hybrids are mostly F_1_ in most rivers. Flannelmouth× white hybrids also include moderate numbers of F_2_ and backcrossed individuals in some locations.

**Figure 6:**
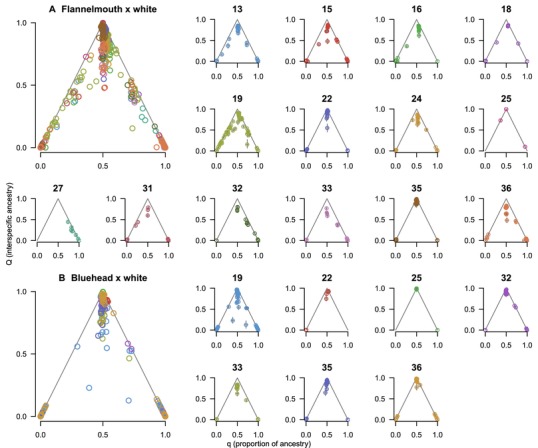
Estimates of proportion of ancestry (*q*) and interspecific ancestry (*Q*) for flannelmouth× white (**A**) and bluehead× white hybrids (**B**) show that the extent of hybridization and backcrossing varies among rivers. The large plots show all hybrids of each cross, color‐coded by river, and the smaller plots each represent one river where hybrids are present. F_1_ hybrids occupy the apex of the gray triangle in each plot (*q*=0.5, *Q*=1). F_2_ hybrids have the same expected proportion of ancestry ($\bar{q}=0.5$), but depressed expectations for interspecific ancestry ($\bar{Q}$=0.5). $BC_{1}$ hybrids have expectations of $\bar{q}$=0.25 or 0.75, and $\bar{Q}$=0.5.

We observed different combinations of *q* (proportion of ancestry) and *Q* (interspecific ancestry) in hybrid individuals in different hybrid crosses, indicating different numbers of generations of hybridization and different parentage of hybrids (Fig. 6). Using *q* and *Q*, we classified hybrid individuals as likely F_1_ (*q* 0.4–0.6; Q>0.75), potential F_2_ (*q* 0.4–0.6; *Q* 0.25–0.75), potential first‐generation backcrosses (*q* 0.15–0.35 or *q* 0.65–0.85; *Q* 0.25–0.75), and other types of hybrids (Fig. 5). Number and identity of hybrid crosses varied by location. Within each hybrid cross, there was variation in what additional hybridization (if any) occurred beyond the F_1_ generation (Figs. 3, 4, 5, 6). In some cases, hybridization led to later‐generation recombinant hybrids and backcrosses; in other cases, outcomes of hybridization were more constrained (asymmetric backcrossing or absence of later generation hybrids).

Flannelmouth× white hybrids were most geographically widespread (Fig. 5, 6). Hybridization ranged from only F_1_ hybrids in some locations (e.g., site 35, the mainstem of the Gunnison in Colorado) to extensive later‐generation hybridization and repeated backcrossing to both parental species (e.g., site 19, Muddy Creek, Carbon County, Wyoming). In some locations, backcrossing was observed only toward one parental species (toward flannelmouth: sites 24, 32, 33, and 36; toward white: sites 16 and 31). In other locations, hybrids were formed through symmetrical backcrossing to both parental species (sites 12, 13, 15, 18, and 19). In contrast, bluehead× white hybrids were mostly first generation (F_1_; Figs. 5, 6), with a small number of backcrosses in several locations (sites 19, 32, and 33) and F_2_ hybrids observed in only one location (site 19).

### ANALYSES OF POSSIBLE CAUSES FOR VARIABLE HYBRIDIZATION

If variation in reproductive isolation is associated with genetic differentiation of populations at a large geographic scale within a species, we expect that hybridization outcomes might be associated with intraspecific genetic structure in one or both parental species. We therefore used principal components analysis to quantify intraspecific population genetic structure and compare to hybridization outcomes for flannelmouth× white sucker hybrids. The range of *q* values (proportion of ancestry; larger range corresponds to more backcrossing) for flannelmouth× white hybrids differed substantially between adjacent localities (Fig. 4, **A**), despite genetic similarity of populations within river basins (proximity in principal component space; see PC1 in Fig. 4 **B** and **C**). There was no correlation between *q* range and PC1 for either parental species (Fig. 4, **B** and **C**), suggesting that extent of hybridization was not correlated with major axes of genetic differentiation within parental species, although it is possible that a small number of causative loci could vary among populations independently of the major axes of variation quantified by a PCA.

If variation in reproductive isolation is connected to ecological context, we expect that hybridization outcomes might be correlated with ecological or environmental characteristics of sites. We used publicly available data quantify the association between attributes of sites (e.g., stream gradient, land usage, elevation) and hybridization outcomes. No environmental attributes of sites were strongly associated with extent of hybridization (more details are included in Supplemental Material). However, the number of parental species at a location was positively and significantly correlated with number of hybrid crosses and extent of backcrossing in flannelmouth× white hybrids (Pearson correlations = 0.56 and 0.51; *P*<0.05). Multiple parental species are needed to produce multiple hybrid crosses, but interestingly, the number of parental species was also positively correlated with extent of backcrossing in a single cross, flannelmouth× white sucker hybridization.

## Discussion

We present evidence for highly variable outcomes of interactions between *Catostomus* species, indicating that reproductive isolation is remarkably inconsistent across the geographic area where these species interact. Furthermore, variation in several pairs of *Catostomus* species suggests that variation in evolutionary interactions between species might be general rather than exceptional (Kozlowska et al. 2012; Fukami 2015). Our findings contribute to the mounting evidence that reproductive isolation and hybridization can vary substantially through space and time, and across different species pairs in secondary contact (Buerkle and Rieseberg 2001; Vines et al. 2003; Lepais et al. 2009; Nolte et al. 2009; Teeter et al. 2010; Haselhorst and Buerkle 2013; Mandeville et al. 2015). In the sections below we elaborate on possible causes and consequences of this variability.

### POTENTIAL CAUSES OF VARIABLE REPRODUCTIVE ISOLATION

Variation in hybridization among populations could stem from variation in loci and traits associated with isolation, variation due to epistatic interactions among genes within individuals, or phenotype‐by‐phenotype interactions between potentially hybridizing individuals. Additionally, variation in hybridization might arise from variation in traits that results from genotype‐by‐environment interactions across heterogenous environments. We lack the evidence to fully evaluate the contributions of these potential causes in the case of *Catostomus* hybridization, but discuss their plausibility below.

Variable genetics underlying reproductive isolation have been described in many taxa (e.g., Wade et al. 1997; Rieseberg 2000; Reed and Markow 2004; Kopp and Frank 2005; Shuker et al. 2005; Vyskočilová et al. 2005; Sweigart et al. 2007; Good et al. 2008; Nolte et al. 2009; Teeter et al. 2010), indicating that genetic components of reproductive isolation can be polymorphic and inconsistent within a species. Variation could arise prezygotically from variable traits in parental taxa, both by simple trait variation or through the interactions of traits of individuals (gene‐by‐gene or phenotype‐by‐phenotype interactions; e.g., matching of phenology). Additionally, variation in isolation could arise postzygotically, from genetically variable progeny that are produced through hybridization (Bomblies and Weigel 2007; Bomblies et al. 2007). If intraspecific variation for genetic components of reproductive isolation is responsible for variation in hybridization (Cutter 2012; Kozlowska et al. 2012), hybridization outcomes could be shared by members of subspecific demes. However, population genetic structure within *Catostomus* parental species was not correlated with outcomes of hybridization (Fig. 4). If variable genetics of reproductive isolation are responsible for variation in *Catostomus* hybridization, the causal alleles are likely to vary at a different spatial scale than the population structure we detected, or not be strongly correlated with major axes of subspecific genetic variation (i.e., differences among sites due to a few loci of large effect rather than overall genomic differentiation).

Genotype‐by‐environment interactions could also produce variable hybridization if genetic and environmental determinants of reproductive isolation, or their efficacy, differ across locations (Seehausen et al. 1997; Taylor and McPhail 2000). For example, in African cichlids, elevated water turbidity can lead to loss of reproductive isolation between sympatric species (Seehausen et al. 1997, 2008). In trout, warming water temperatures have led to increased hybridization between native and nonnative species (Muhlfeld et al. 2014). It is also likely that time since introduction of a nonnative species or proximity to introduction site could affect hybridization outcomes (as in tiger salamanders; Fitzpatrick et al. 2010), but since the introduction history of white sucker populations is poorly characterized, it is difficult to know how much introduction history affects hybridization outcomes. However, adjacent populations sometimes have different hybridization outcomes despite probable similarity in introduction times, suggesting that time since introduction is not the primary determinant of extent of hybridization.

### EVOLUTIONARY SIGNIFICANCE OF VARIABLE REPRODUCTIVE ISOLATION

Research on hybridization and speciation has typically focused on processes that maintain reproductive isolation for species as a whole (Endler 1977; Barton and Hewitt 1985; Hewitt 1988; Barton and Hewitt 1989). In contrast, our study of *Catostomus* hybridization indicates that realized reproductive isolation is extremely variable across many locations, among several pairs of *Catostomus* fishes, which implies that no single, consistent set of mechanisms is responsible for maintaining reproductive isolation between these species. Along with other studies, our findings support the idea that variability in isolating barriers might be common for incompletely isolated and potentially hybridizing taxa (Sweigart et al. 2007; Good et al. 2008; Kozlowska et al. 2012). This variation exists in the context of likely secondary contact among *Catostomus* species, whereas variation might exist in other systems that have undergone primary divergence, among populations that have evolved isolation to different extents (Riesch et al. 2017; Stuart et al. 2017).

For effective reproductive isolation to exist at all geographic locations where *Catostomus* species co‐occur would require different natural selection among sites. At locations with primarily F_1_ hybrids, effective isolation would arise from selection against the traits of F_1_ hybrids (e.g., low F_1_ fecundity). In contrast, at sites with relatively high F_1_ fitness (as in trout and salamanders; Fitzpatrick and Shaffer 2007; Muhlfeld et al. 2009; Fitzpatrick et al. 2010) and viable later‐generation F_*n*_ and backcrossed hybrids, effective isolation would instead require very low fertility of F_*n*_ and backcrossed hybrids. However, beyond the F_1_, or if F_1_ hybrids themselves are variable, hybridization produces a broad range of genotypes and phenotypes, rather than a single hybrid phenotype (Gompert and Buerkle 2016). We do not have direct observations of the fitness of *Catostomus* hybrids. If hybridization proceeds beyond the F_1_, it is less likely that selection would effectively maintain isolation, and local gene flow between species would be likely (Barton and Bengtsson 1986; Gavrilets and Gravner 1997; Bank et al. 2012; Gompert et al. 2012; Lindtke and Buerkle 2015). Adaptive and neutral introgression of traits and genomic regions across species boundaries is also likely to vary geographically, potentially affecting local evolutionary and ecological dynamics. It is unclear what long‐term outcomes of variable hybridization and introgression will be in *Catostomus* fishes, and more generally, what consequences heterogeneity in species boundaries has for the evolutionary cohesion of populations within parental species.

### ECOLOGICAL CONSEQUENCES OF VARIABLE REPRODUCTIVE ISOLATION

If variable postzygotic selection on hybrids drives variation in hybridization among sites, ecological traits of hybrids will be important to outcomes of interactions between species. Fitness of hybrids might therefore vary geographically as a result of variation in ecologically important traits. Hybrids often have different phenotypes from either parental species, and might be able to exploit different resources (e.g., Williams and Ehleringer 2000; Lexer et al. 2004; Gompert et al. 2006; Rieseberg et al. 2007; Stelkens and Seehausen 2009). Specific to this system, we know that different *Catostomus* species have different diets and swimming abilities (Cross et al. 2013; Walsworth et al. 2013; Underwood et al. 2014), and overlap in spawning habitat and timing to varying extents (Sweet and Hubert 2010). Given their variation in admixture, and the different crosses involved, *Catostomus* hybrids are likely to express a wide range of phenotypes, perhaps including transgressive phenotypes (Stelkens and Seehausen 2009; Stelkens et al. 2009). In locations with more extensive backcrossing or later‐generation hybridization, recombinant hybrids with high fitness might be formed, potentially with novel ecological traits (as in sunflowers; Rieseberg et al. 2003) or simply highly competitive relative to the parental species, leading to variable ecological outcomes of hybridization.

The patterns of hybridization described in this study are important for conservation of native *Catostomus* species in the Upper Colorado River basin. Our conclusions from this study also apply more generally to understanding when extinction via hybridization is likely to occur. Two species in this study, *C. discobolus* and *C. latipinnis*, are the focus of conservation and management in Wyoming, Colorado, and Utah (Gill et al. 2007; Gelwicks et al. 2009; Senecal et al. 2010), and population sizes are believed to be declining (Bezzerides and Bestgen 2002). Hybridization with nonnative *C. commersoni* has been viewed as a major threat to persistence of native species. Initially, based on results of McDonald et al. (2008), the primary concern for management was the potential loss of genetic identity of native taxa. However, based on results reported here and previously (Mandeville et al. 2015), it is likely that genetic homogenization of native and nonnative species will occur locally, if at all. Hybridization would lead to introgression in some locations, blurring genetic identity of parental species (Rhymer and Simberloff 1996; Wolf et al. 2001), but local genetic homogenization of these species would be unlikely in locations with little hybridization or no hybridization beyond the F_1_ (Fig. 3,5). Identifying variation in effectiveness of reproductive isolation will help managers prioritize where and how to intervene. Demographic threats to the persistence of native species also exist and might be exacerbated by variation in ecological success of hybrids. Hybridization very likely results in an opportunity cost and lower population mean fitness, since heterospecific reproduction almost certainly occurs at the cost of conspecific reproduction. The question for management is how large this cost is. If the reduction in population mean fitness is large, then hybridization of any of the forms we observed might represent a substantial threat to the local persistence of species. If mean fitness is reduced only slightly as a result of hybridization, introgression and locally homogenizing gene flow would be a greater concern.

### CONCLUSIONS

In this study, we provide evidence for variable genomic outcomes of hybridization among multiple *Catostomus* species pairs across a large geographic area. The variation we observed in hybridization suggests that reproductive isolation is also variable, with no single mechanism of reproductive isolation maintaining separation between species across all locations where they come into contact. Few studies have examined outcomes of hybridization across a similar geographic area. Thus, it is unclear to what extent our results represent a general pattern of variable reproductive isolation and variable evolutionary consequences of species interactions. It is possible that the variation we observed is characteristic of reproductive isolation in many taxa. Variation in outcomes of reproductive interactions between species would be consistent with what we know about the ecological outcomes of interactions between species, which are more commonly recognized to be influenced by contingency and context than are primarily evolutionary outcomes of species interactions like hybridization. If *Catostomus* fishes are indeed representative of typical dynamics of reproductive isolation across the range of an interaction between species, this suggests that our conceptual models of reproductive isolation as a consistent process operating at the species level might need to be revised to accommodate potential for substantial variation across time and space.

Associate Editor: Rhonda Snook

## Supporting information


**Table S1**: Locations sampled in this study.
