# Supplementary material for: Inconsistent reproductive isolation revealed by interactions between Catostomus fish species
Source: Evol Lett. 2017 Oct 27;1(5):255–68. doi: 10.1002/evl3.29 (PMC6121845; doi:10.1002/evl3.29)
Supplement: Supplementary file 1 — Table S1: Locations sampled in this study. [file EVL3-1-255-s001.zip › evl329-sup-0001-SuppMat.pdf]

Supplemental Analyses

Methods and Results: Environmental variables do not predict hybridization outcomes

We used a large environmental dataset to examine relationships between environmental variables and hybridization outcomes. Environmental data were assembled by S. Albeke (WYGISC, University of Wyoming). Data come from public sources, including the National Hydrography Dataset (1:24,000 scale), STATSGO (soil data), EPA Ecoregion data, PRISM data, and state geological survey data for Wyoming and Colorado. Each environmental variable is a point estimate at the location where fish were collected. Some environmental variables are also quantified at the basin level. Our environmental response variables included land type and land use variables for the surrounding watershed (vegetation type, agricultural activity, human use), precipitation, elevation, basin gradient, sediment characteristics, and geologic attributes of the surrounding land. These environmental characteristics could potentially be associated either with prezygotic barriers to hybridization (e.g. habitat partitioning, spawning phenology, mate recognition) or postzygotic selection on hybrids.

We used `cor.test` in R to test for correlations between environmental variables and the hybridization outcomes quantified by our two response variables. We quantified extent of hybridization with two different variables, each of which captures a different dimension of hybridization. Multiple hybridization metrics were required because there are several ways that a fish assemblage can have extensive hybridization: 1) many different crosses involving multiple species, 2) extensive hybridization beyond F1 within a single cross, or 3) both many crosses and extensive hybridization beyond F1 hybrids. Hybridization response variables were 1) number of hybrid crosses and 2) range of q for flannelmouthwhite hybrids (the most geographically widespread hybrid combination), corresponding to the extent of backcrossing in this cross. We calculated pairwise correlations between hybridization variables and each environmental variable. Since multiple statistical comparisons were made, we applied a sequential Bonferroni method (Holm-Bonferroni in R) to adjust p-values and correct for potential Type I errors. After correcting for multiple statistical comparisons, no environmental variables were significantly associated with hybridization outcomes.

Supplemental Table

Site code	Site name	HUC2	HUC2 name	Basin
1	Jackson Lake	17	Pacific Northwest	Snake
2	Pacific Creek	17	Pacific Northwest	Snake
3	Snake River	17	Pacific Northwest	Snake
4	Black Rock Creek	17	Pacific Northwest	Snake
5	Spread Creek	17	Pacific Northwest	Snake
6	Granite Ditch	17	Pacific Northwest	Snake
7	Gros Ventre River	17	Pacific Northwest	Snake

8	Hoback River	17	Pacific Northwest	Snake
9	Fremont Lake	14	Upper Colorado	Green
10	North Horse Creek	14	Upper Colorado	Green
11	Half Moon Lake	14	Upper Colorado	Green
12	Big Sandy River	14	Upper Colorado	Green
13	Little Sandy Creek	14	Upper Colorado	Green
14	Bitter Creek	14	Upper Colorado	Green
15	Black's Fork	14	Upper Colorado	Black's Fork
16	Green River (WY)	14	Upper Colorado	Green
17	Littlefield Creek	14	Upper Colorado	Yampa
18	Muddy Creek	14	Upper Colorado	Black's Fork
19	Muddy Creek	14	Upper Colorado	Yampa
20	Ringdahl Reservoir	14	Upper Colorado	Green
21	Henry's Fork	14	Upper Colorado	Henry's Fork
22	Little Snake (WY)	14	Upper Colorado	Yampa
23	Little Snake (CO)	14	Upper Colorado	Yampa
24	Yampa River	14	Upper Colorado	Yampa
25	Williams Fork	14	Upper Colorado	Yampa
26	McKinney Creek	14	Upper Colorado	Yampa
27	Kenney Reservoir	14	Upper Colorado	White
28	Piceance Creek	14	Upper Colorado	White
29	Coal Creek	14	Upper Colorado	White
30	White River (Meeker)	14	Upper Colorado	White
31	Green River (UT)	14	Upper Colorado	Green
32	Colorado River (Clifton)	14	Upper Colorado	Colorado
33	Colorado River (Grand Junction)	14	Upper Colorado	Colorado
34	East Creek	14	Upper Colorado	Gunnison
35	Gunnison	14	Upper Colorado	Gunnison
36	Escalante Creek	14	Upper Colorado	Gunnison
37	Roubideau Creek	14	Upper Colorado	Gunnison
38	Potter Creek	14	Upper Colorado	Gunnison
39	San Miguel River	14	Upper Colorado	Dolores
40	Tabaguache Creek	14	Upper Colorado	Dolores
41	Dolores River	14	Upper Colorado	Dolores
42	Mancos River	14	Upper Colorado	San Juan
43	San Juan (Farmington)	14	Upper Colorado	San Juan
44	Long Hollow Creek	14	Upper Colorado	San Juan
45	La Plata River	14	Upper Colorado	San Juan
46	Nenahnezad	14	Upper Colorado	San Juan
47	Bear River	16	Great Basin	Bear
48	Little Goose Creek	10	Missouri	Powder
49	North Redwater Creek	10	Missouri	Cheyenne
50	Bighorn River	10	Missouri	Bighorn
51	Buffalo Bill Reservoir	10	Missouri	Bighorn
52	Big Goose Creek	10	Missouri	Powder

53	LAK Reservoir	10	Missouri	Cheyenne
54	East Fork Wind River	10	Missouri	Bighorn
55	Little Wind River	10	Missouri	Bighorn
56	Popo Agie	10	Missouri	Bighorn
57	Sweetwater River	10	Missouri	North Platte
58	Laramie River	10	Missouri	North Platte
59	North Crow Reservoir	10	Missouri	North Platte
60	Boulder Creek	10	Missouri	South Platte
61	Monument Creek	11	Arkansas	Arkansas

Table S1: Locations sampled in this study. Numeric codes correspond to Fig. 1 and are used throughout the text.
